# Polymorphisms and pharmacogenomics for the toxicity of methotrexate monotherapy in patients with rheumatoid arthritis: A systematic review and meta-analysis

**DOI:** 10.1097/MD.0000000000006337

**Published:** 2017-03-24

**Authors:** Qi Qiu, Jing Huang, Yang Lin, Xiaoming Shu, Huizheng Fan, Zhihua Tu, Youwen Zhou, Cheng Xiao

**Affiliations:** a Institute of Clinical Pharmacology, Beijing Anzhen Hospital, Capital Medical University; b Institute of Clinical Medicine, China-Japan Friendship Hospital; c Beijing University of Chinese Medicine; d Department of Rheumatology, China-Japan Friendship Hospital, Beijing; e Department of Gastroenterology, People's Hospital of Yichun, Jiangxi Yichun; f Department of Rheumatology, Yili Kazak Autonomous Prefecture Hospital of Traditional Chinese Medicine, Xinjiang Yining, China; g Department of Dermatology and Skin Science, University of British Columbia; h Molecular Medicine Lab and Chieng Genomics Center, Vancouver Coastal Health Research Institute, Vancouver, BC, Canada.

**Keywords:** meta-analysis, methotrexate, pharmacogenomics, polymorphisms, rheumatoid arthritis, systematic review, toxicity

## Abstract

**Background:**

Methotrexate (MTX) is widely used and considered a first-line disease modifying antirheumatic drug (DMARD) for the treatment of rheumatoid arthritis (RA). However, 10% to 30% of patients discontinue therapy within a year of starting the treatment, usually because of undesirable side effects. Many of the relevant genes have been investigated to estimate the association between gene polymorphisms and MTX toxicity in RA patients, although inconsistent results have been reported.

**Methods:**

We searched EMBASE and PubMed in February 2016 for polymorphisms and pharmacogenomics study of the toxicity of MTX monotherapy in RA patients. The meta-analysis was stratified by whether genetic variants associated with MTX toxicity.

**Results:**

A total of 42 publications that included 28 genes with 88 gene SNPs associated with the transporters, enzymes, and metabolites of MTX or the progression of RA were included in the SR, and 31 studies were included in 7 meta-analyses. The meta-analysis showed a significant association between the toxicity of MTX and the RFC-1 80G > A (rs1051266) polymorphism in the European RA patients.

**Conclusion:**

RFC-1 80G > A (rs1051266) polymorphism was associated with MTX toxicity, and larger and more stringent study designs may provide more accurate results for the effect of these SNPs on the MTX toxicity.

## 1 Introduction

Rheumatoid arthritis (RA) is a systemic autoimmune disease characterized by chronic synovial joint inflammation, which leads to disability and diminished quality of life.^[1,2]^ The main objectives for managing RA are to control pain, prevent or control joint damage, and avoid long-term loss of function. Disease-modifying antirheumatic drugs (DMARDs) are mainstay treatments for controlling the symptoms of RA and modifying its radiographic progression.^[3]^ There are several DMARDs available; however, since the reintroduction of methotrexate (MTX) in the early 1980s, MTX has become the most highly effective, fast-acting, disease-modifying antirheumatic drug and is one of the most widely used and the first-line DMARD for the treatment of RA.^[4,5]^ Accumulating evidence has indicated that earlier treatment with DMARD therapy improves long-term outcomes.^[3,6,7]^

Although the combined efficacy and continuation rates for MTX are superior to that of other DMARDs.^[3]^ Responses to MTX in terms of both efficacy and toxicity vary considerably between patients implying the necessity to study factors that may contribute to such interindividual variability.^[8]^ Estimates indicated that in 10% to 30% of the patients, MTX therapy is discontinued because of adverse effects.^[9,10]^ Various factors, including individual patient factors, disease-specific factors, and genetic factors, have been shown to influence the toxicity of MTX. Therefore, consistently reliable clinical or molecular markers are not available to accurately predict the response to MTX therapy. Pharmacogenomics refers to the study of the entire genome (covering transcriptomic and proteomic fields) and the expression levels of individual genes (mRNA) to identify the genetic factors influencing adverse effects and toxicity to MTX treatment.^[11]^ Researchers believe that pharmacogenetic markers may offer a strategy to help identify patients who are more likely to suffer the toxicity of MTX, although this hypothesis requires clinical evidence.

The reasons behind patient occurrence of adverse events remain unclear, but research into these issues has generated considerable interest. Many of the relevant genes involved in the metabolism of MTX have been investigated to estimate the association between gene polymorphisms and MTX toxicity in RA patients.^[4]^ However, these studies have produced mixed results because of their small sample size and poor statistical power. A meta-analysis can provide a potential solution to this problem because these evaluations combine the results from several studies. Indeed, one of the major advantages of using meta-analyses is the ability to evaluate larger sample sizes, which reduces the likelihood of random errors producing false-positive or false-negative associations. Therefore, to overcome the limitations of individual studies, resolve inconsistencies, and increase precision, we performed a meta-analysis in our study to determine whether the gene polymorphisms in the evaluated studies can predict the adverse events or toxicity to MTX therapy in patients with RA.

Over the past 10 years, 7 meta-analyses^[1,4,12–16]^ on the association between polymorphisms and the toxicity of MTX in RA patients were published in the PubMed and Embase databases. To the best of our knowledge, this is the first systematic review (SR) summarizing all of the available studies on the association between single nucleotide polymorphisms (SNPs) and responsiveness to MTX in RA patients. In the present study, we focused on studies that reported the toxicity of MTX monotherapy and utilized pharmacogenetics, or the analysis of an individual's genetic variations to predict MTX toxicity in treatment. We also updated the meta-analysis of the MTHFR (677C > T (rs1801133) and 1298A > C (rs1801131)), ABCB1 3435C > T (rs1045642), RFC-1 80G > A (rs1051266), and ATIC 347C > G (rs2372536) polymorphisms and completed the first meta-analysis on the association between MTR A2756G (rs1805087) and MTRR 66A > G (rs1801394) SNPs and the toxicity of MTX in RA patients and the MTHFR (677C > T (rs1801133) and 1298A > C (rs1801131)) and RFC-1 80G > A (rs1051266) polymorphisms were included in the homology subgroup analysis.

## 2 Methods

The methodology for this study was based on the Preferred Reporting Items for SRs and Meta-Analyses (PRISMA) statement.^[17]^ Ethical approval was not necessary for this meta-analysis because the results included pooled data from individual studies that received ethics approval.

### 2.1 Published study identification and selection for meta-analysis

**Figure 1:**
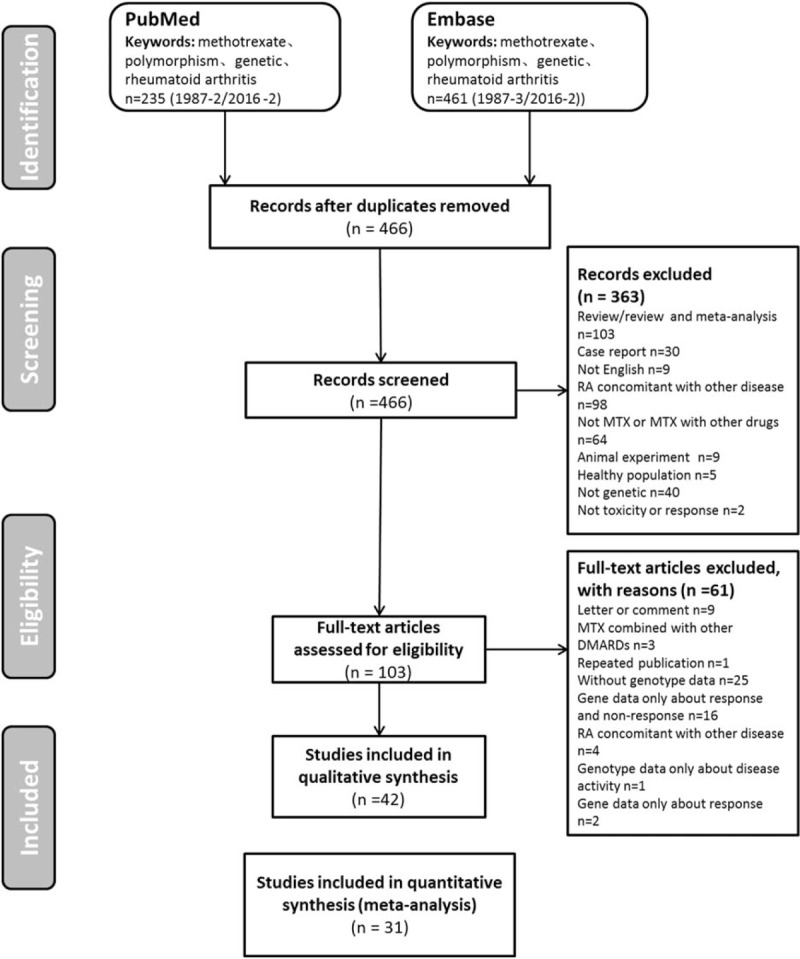
Study selection flow diagram adapted from the Preferred Reporting Items for SRs and Meta-Analyses (PRISMA) Statement.

All studies investigating the relationship between SNPs and MTX toxicity in RA published before February 2016 were identified using computer-based searches of the PubMed database and Embase database (OvidSP) using the following combination of keywords ‘methotrexate[Title/Abstract] AND (polymorphism[Title/Abstract] OR polymorphisms[Title/Abstract] OR genetic[Title/Abstract])) AND (“arthritis, rheumatoid”[MeSH Terms] OR (“arthritis”[All Fields] AND “rheumatoid”[All Fields]) OR “rheumatoid arthritis”[All Fields] OR (“rheumatoid”[All Fields] AND “arthritis”[All Fields]))’. Details of the search flow are provided in Fig. 1. The titles alone were initially reviewed for suitability, and then the abstracts of these titles were obtained and reviewed to determine the full-text retrieval suitability. Data were then extracted as described in the following section from suitable full-text reports. Only studies of human subjects that used validated genotyping methods were included. Case reports, editorials, and review articles were excluded.

### 2.2 Data extraction

References were screened and data were extracted independently by 2 authors (QQ and JH) using a predetermined data collection template. To resolve discrepancies on the inclusion of studies and interpretation of data, a third investigator (YL) was consulted, and consensus was reached by discussion. The following data were recorded: first author's last name, year of publication, location of study, inclusion and exclusion criteria, sample size, MTX dose, SNP analysis results, treatment duration, and demographic details of patients, follow-up period, toxicity criteria and adverse events.

### 2.3 Statistical analyses

The gene SNPs detected in more than 2 studies were included in the meta-analysis. Genotype frequencies for the MTHFR (677C > T (rs1801133) and 1298A > C (rs1801131)), RFC-1 80G > A (rs1051266), ATIC 347C > G (rs2372536), MTR A2756G (rs1805087), MTRR 66A > G (rs1801394), and ABCB1 3435C > T (rs1045642) polymorphisms were determined. We examined the differences in CC versus (CT + TT) for the MTHFR 677C > T (rs1801133) and ABCB1 3435C > T (rs1045642) polymorphisms; AA versus (AC + CC) for the MTHFR 1298A > C (rs1801131) polymorphism; GG versus (GA + AA) for the RFC-1 80G > A (rs1051266); CC versus (CG + GG) for the ATIC 347C > G (rs2372536) polymorphism; and AA versus (AG + GG) for the MTRR 66A > G (rs1801394) polymorphism. This process corresponded to a dominant model that assumes a dominant effect of the minor allele, which is consistent with a previous meta-analysis and allowed for the inclusion of a maximum number of studies.^[9,14,15]^ For each study, the point estimate of risk, the OR, and the corresponding 95% CIs of MTX with AE versus without AE were calculated. Then, the overall pooled OR and corresponding 95% CIs were estimated using the Mantel–Haenszel method, and the fixed effect was the absence of moderate inconsistency (>50%) across studies.^[3]^ A fixed effect framework assumes that the effect of allele frequency is constant across studies and between-study variations are caused by chance or random variation. The random effects model was used when heterogeneity > 50%, and it assumes different underlying effects, considers both within- and between-study variation, and is advantageous because it accommodates diversity between studies and provides a more conservative estimate. The odds ratio (OR) was pooled using inverse variance methods to generate a summary OR and 95% confidence interval (CI). We assessed the heterogeneity between the included studies using the *χ*^2^-based Cochran Q statistic. The percentage of across-study variability attributable to heterogeneity beyond chance was estimated using the *I*^2^ statistic. Differences in the pooled ORs were compared using a *Z* test. A 2-sided *P* value of less than 0.05 was considered significant for all analyses. All statistical meta-analyses were completed with STATA (version 13.0; Stata Corp, College Station, TX).^[18]^ The quantitative results are expressed as mean ± SD.^[19,20]^

## 3 Results

### 3.1 Study selection

Figure 1 shows the study selection process. The initial search identified 696 publications (PubMed: 235; and Embase: 461). The full text of 103 articles was reviewed in detail, and 61 articles were further excluded for the following reasons: letter or comment (n = 9), MTX combined with other DMARDs (n = 3), repeated publication (n = 1), without genotype data (n = 25), gene data only about response and nonresponse (n = 16); RA concomitant with other disease (n = 4); genotype data only about disease acitivity (n = 1); and gene data only about response (n = 2). Ultimately, 42 publications were included in the SR and 31 studies were included in 7 meta-analyses.

### 3.2 Study characteristics

**Figure 2:**
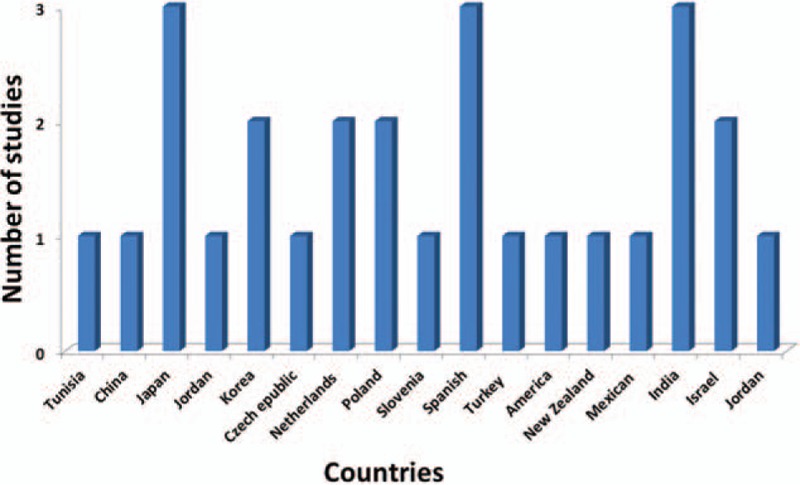
Distribution of countries in 31 studies that measured the association between polymorphisms and the toxicity to MTX in RA. MTX = methotrexate, RA = rheumatoid arthritis.

**Figure 3:**
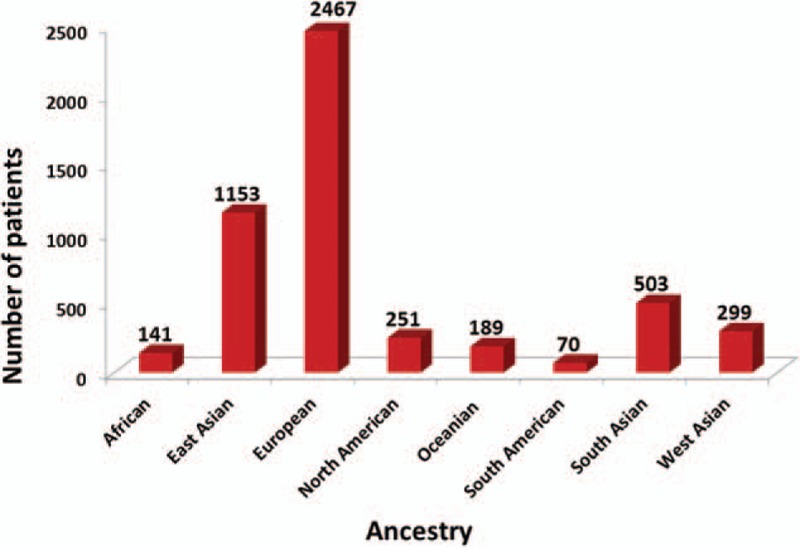
Distribution of ancestry in 31 studies that measured the association between polymorphisms and the toxicity to MTX in RA. MTX = methotrexate, RA = rheumatoid arthritis.

For the analyzed studies, the characteristics and detected genes are shown in Table 1     . The number of papers from Europe accounts for a large proportion of the total number of papers (Figs. 2 and 3).

**Table 1:**
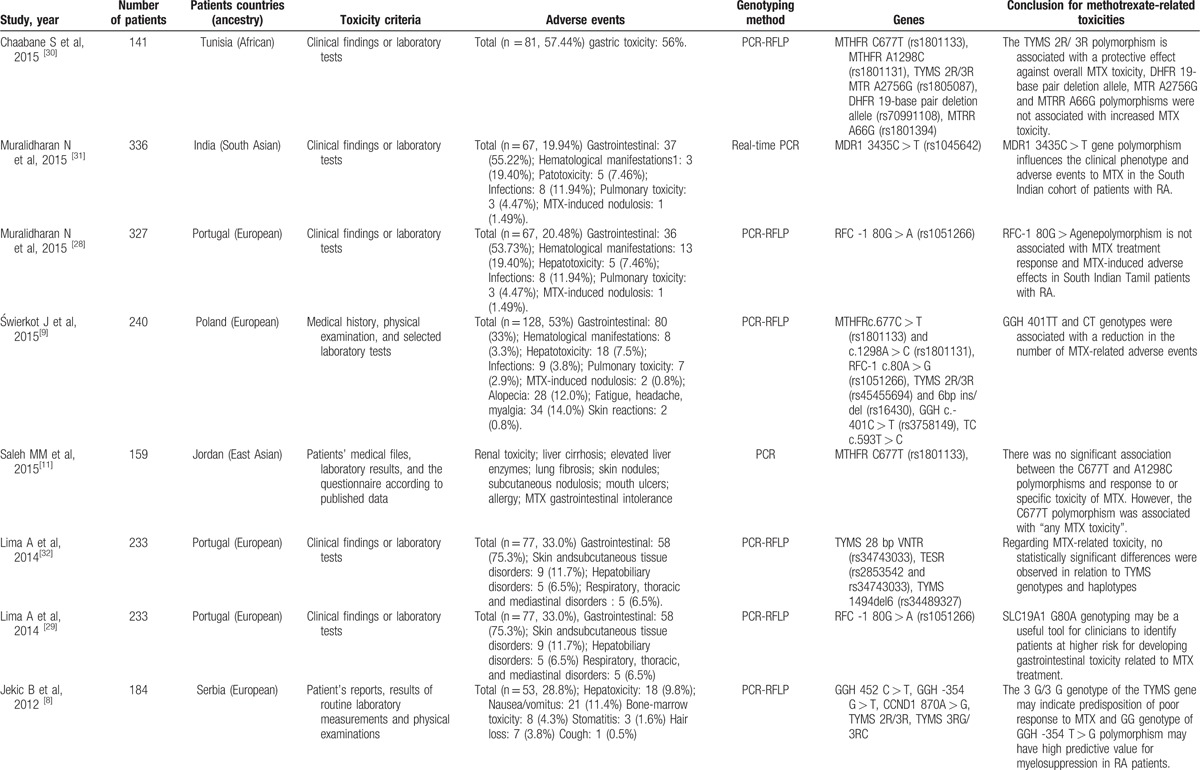
Studies reporting methods of associating polymorphisms with toxicity to MTX in RA.

**Table 1 (Continued):**
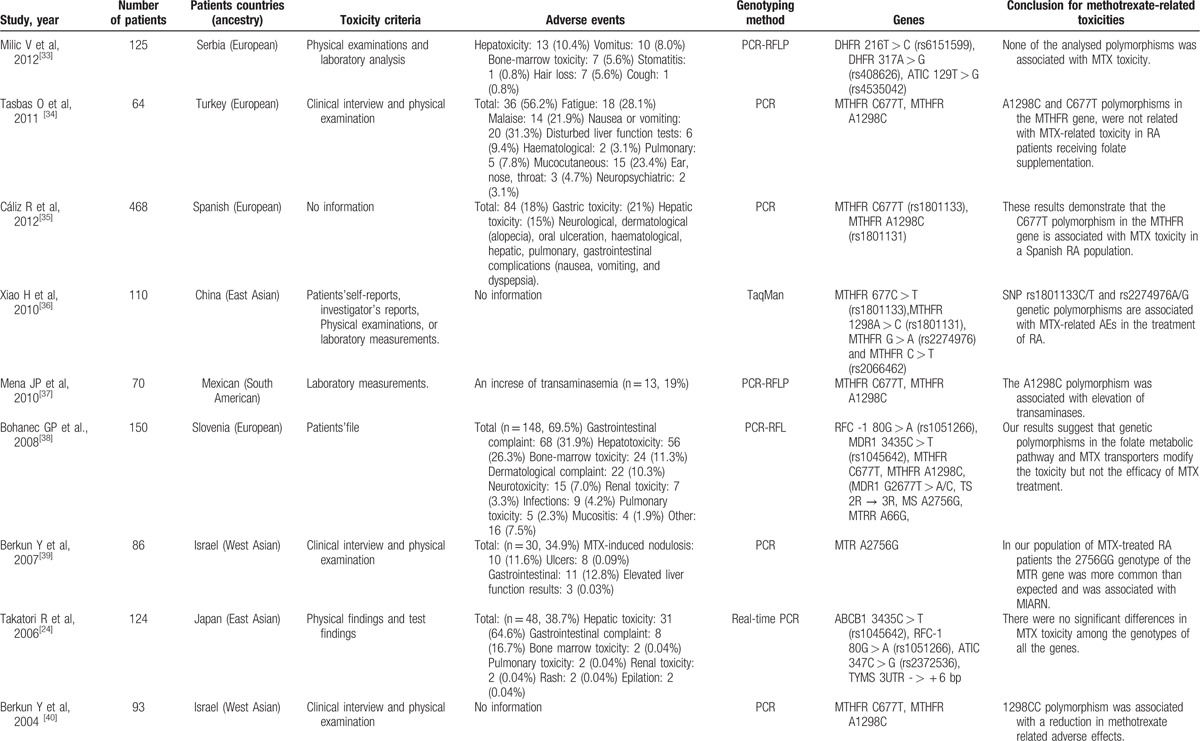
Studies reporting methods of associating polymorphisms with toxicity to MTX in RA.

**Table 1 (Continued):**
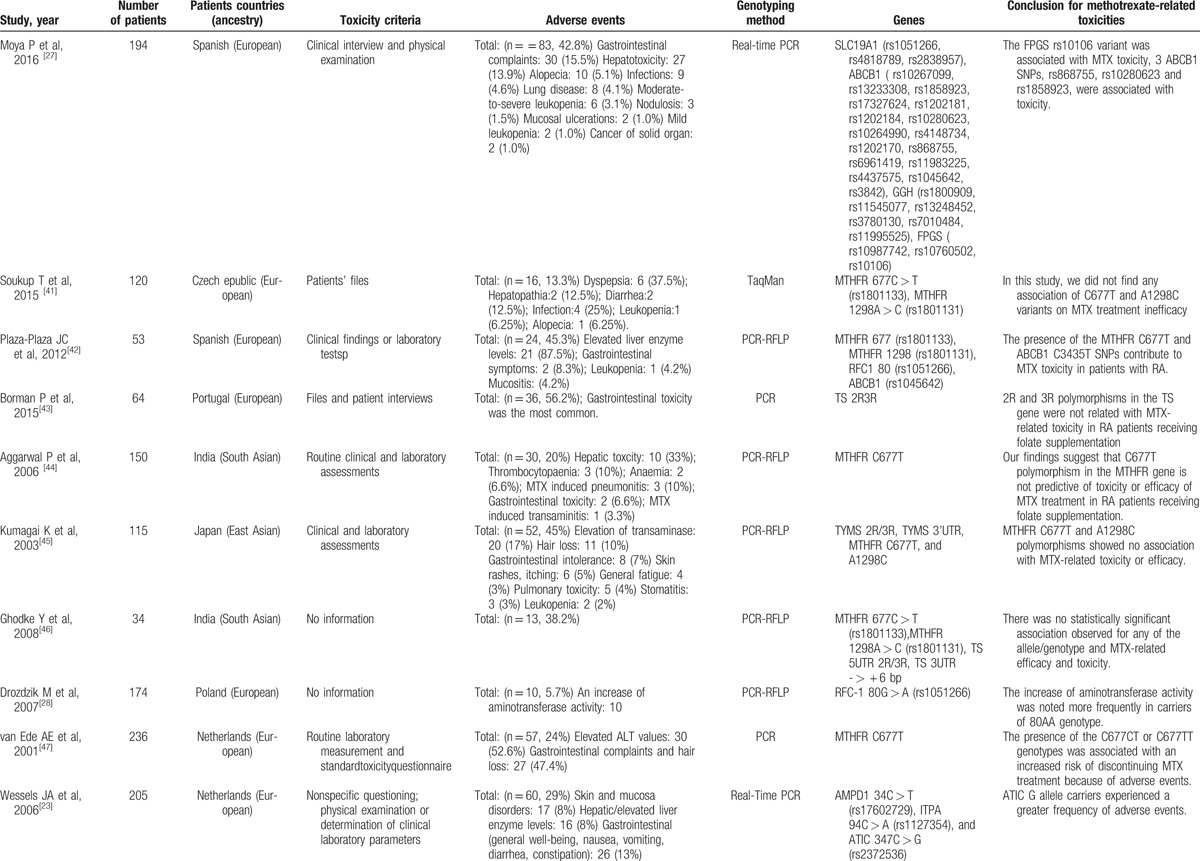
Studies reporting methods of associating polymorphisms with toxicity to MTX in RA.

**Table 1 (Continued):**
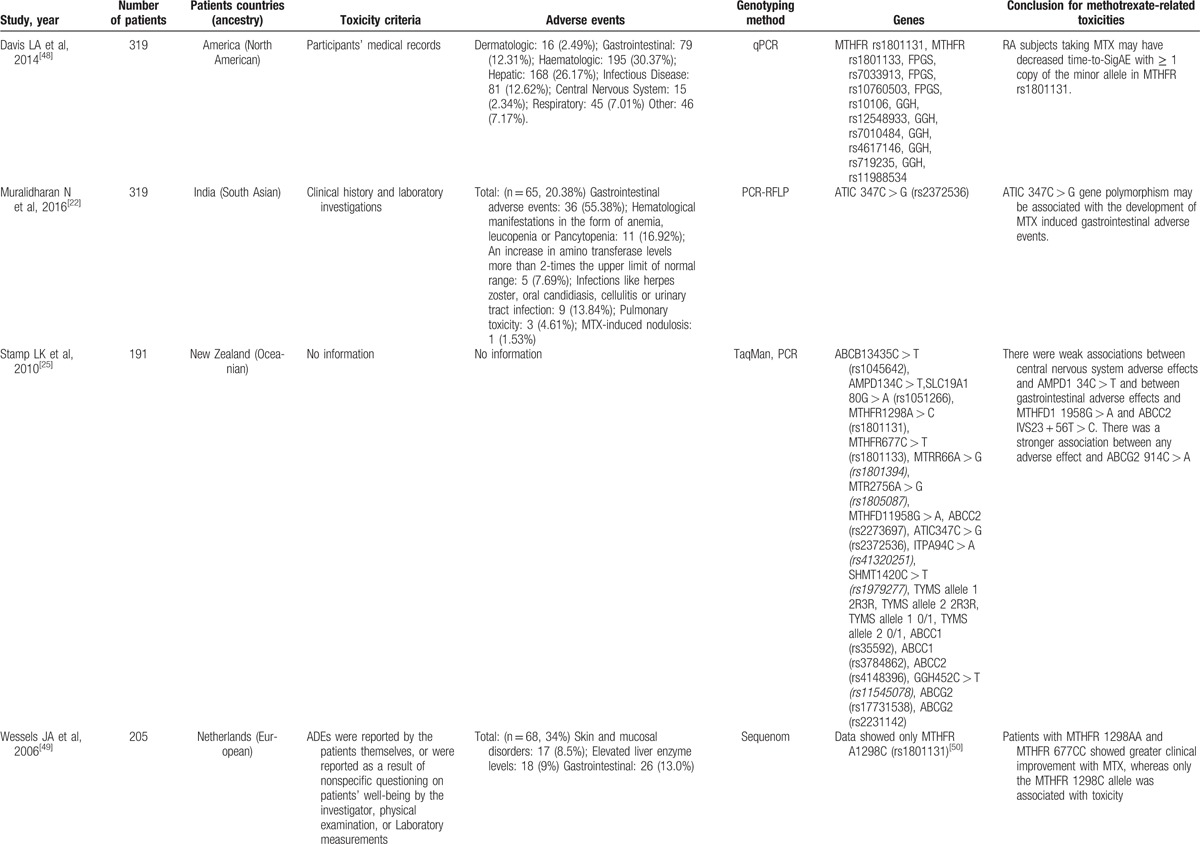
Studies reporting methods of associating polymorphisms with toxicity to MTX in RA.

**Table 1 (Continued):**
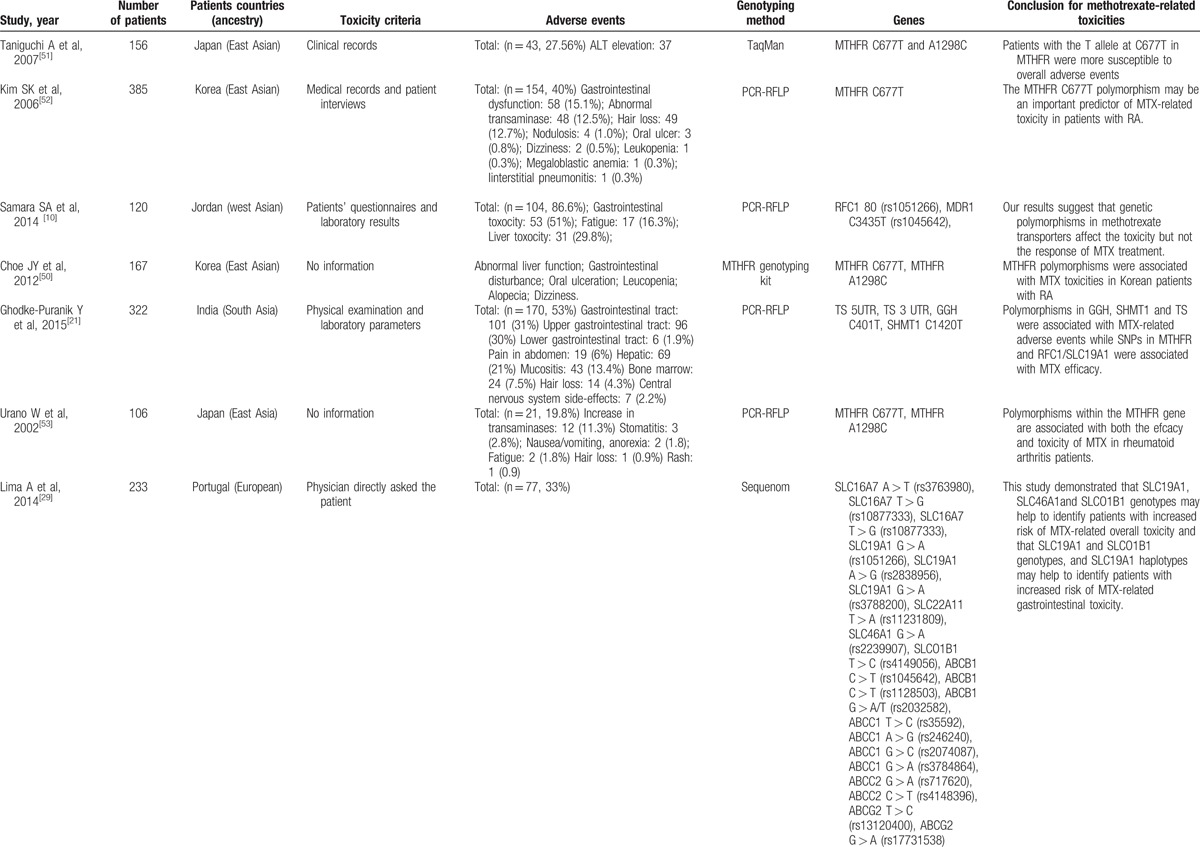
Studies reporting methods of associating polymorphisms with toxicity to MTX in RA.

**Table 1 (Continued):**
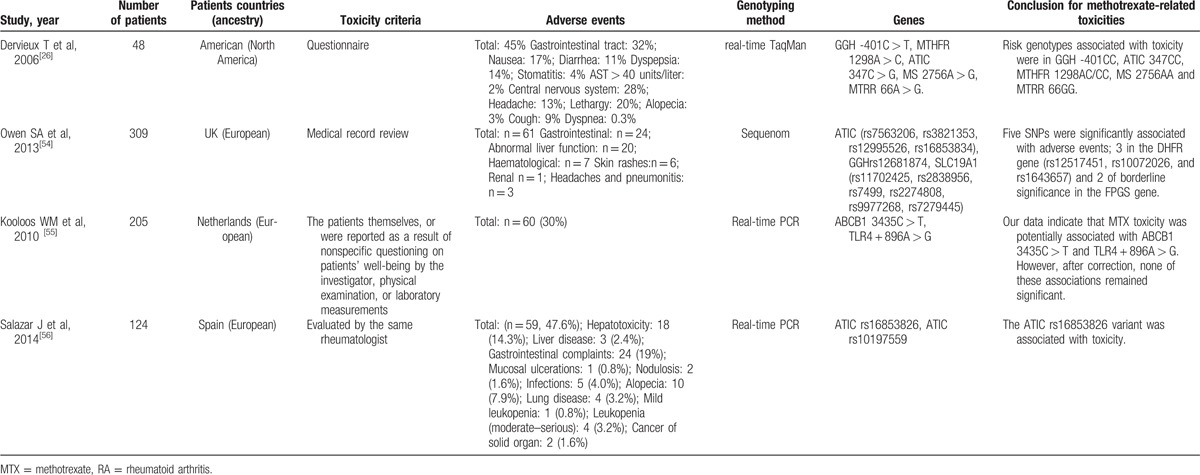
Studies reporting methods of associating polymorphisms with toxicity to MTX in RA.

### 3.3 Pharmacogenetic markers of RA response to MTX treatment

A total of 28 genes with 88 gene SNPs associated with the transporters, enzymes, and metabolites of MTX or the progression of RA were evaluated to explore the association between the gene polymorphisms and the MTX toxicity in previous studies (Table 1     ).

The main action of MTX is to inhibit the folate pathway and exert anti-inflammatory and anti-proliferative effects in RA. The present researches of the MTX metabolic pathway showed that MTX enters target cells through reduced folate carriers (SLC19A1 (RFC-1)) and effluxes from target cells through ATP-binding cassettes (ABCs), predominantly ABCC1–2, ABCB1, and ABCG2.^[57]^ After polyglutamated by the enzyme FPGS, the polyglutamated MTX (MTX-PG) can be reversed by the enzyme GGH, and retained within the cells. The MTX-PG can inhibit the activity of DHFR competitively and reduce the dihydrofolation of tetrahydrofolate (THF), which is the precursor of the biologically active folate cofactor 5-methyl-THF, and this conversion is catalyzed by MTHFR. MTHFR, SHMT, and other enzymes in 1 carbon pool (MS, MTR, and MTRR) are not directly inhibited by MTX, although their expression level may contribute to the antifolate effects of MTX through subtle alterations in the folate pools.^[21,58]^ MTX-PG can inhibit the TYMS (TSER)-mediated conversion of deoxyuridylate to deoxythymidylate in the de novo pyrimidine biosynthetic pathway and can also inhibit the activity of the enzyme ATIC and promote the intracellular accumulation of adenosine (AICAR), which, through a series of enzymatic reactions, leads to the generation of adenosine and increased extracellular concentrations of adenosine, an anti-inflammatory agent. This pathway includes the intermediates inosine monophosphate and inosine triphosphate and the key enzymes ITPA, IMP (IMPDH), and AMP (AMPD1 and ADA). CCND1 controls cell progression through the G1/S phase and is also involved in the regulation of TYMS (TSER) and DHFR.

**Figure 4:**
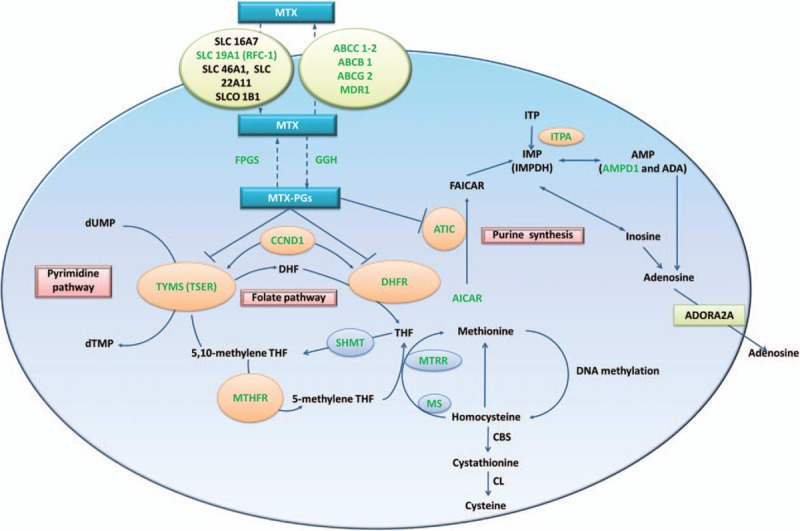
Summary of detected genes associated with the MTX toxicity in RA patients in previous studies. Schematic representation of the intracellular folate biosynthetic pathway and the genes detected in previous studies (in green). ABCC1–4, ABCB1, and ABCG2 = adenosine triphosphate-binding cassette (ABC) transporters, ADA = adenosine deaminase, ADP = adenosine diphosphate, AICAR = 5-aminoimidazole-4-carboxamide ribonucleotide, AMP = adenosine monophosphate, ATIC = -aminoimidazole-4-carboxamide ribonucleotide transformylase/IMP cyclohydrolase, ATP = adenosine triphosphate, CBS = cystathionine-β-synthase, CCND1 = cyclin D1, CH3 = methyl group, CL = cystathionine lyase, DHF = dihydrofolate, DHFR = dihydrofolate reductase, dTMP = deoxythymidine-5′-monophosphate, dUMP = deoxyuridine-5′-monophosphate, FAICAR = 10-formyl-AICAR, FPGS = folylpolyglutamyl synthase, GGH = glutamyl hydrolase, IMP = inosine monophosphate, IMPDH2 = inosine 5′-monophosphate dehydrogenase, ITP = inosine triphosphate, ITPA = inosine triphosphate pyrophosphatase, MDR1 = multidrug resistance 1, MS = methionine synthase, MTHFD1 = methylenetetrahydrofolate dehydrogenase, MTHFR = methylenetetrahydrofolate reductase, MTR = methionine synthase, MTRR = methionine synthase reductase, MTX = methotrexate, MTX-PGs = methotrexate polyglutamates, RFC-1 = reduced folate carrier 1, SHMT = serine hydroxymethyltransferase, SLC16A7, SLC19A1, SLC46A1, and SLC22A11 = solute carriers, SLCO 1B1 = solute carrier organic anion transporter, THF = tetrahydrofolate, TSER = thymidylate synthase enhancer region, TYMS = thymidylate.

The aforementioned genes are commonly used as important candidate genes in studies of RA response to MTX treatment. All of the genes and pathways included in the present SR are summarized in Fig. 4, where they are highlighted in green.

### 3.4 MTHFR 677C > T (rs1801133)

Twenty studies were included in the meta-analysis of MTHFR 677C > T (rs1801133), which contained data from a combined total of 1330 patients with adverse event (AE) and 1941 patients without AE and included 7 European studies (433 patients with AE and 897 patients without AE), 6 East Asian studies (577 patients with AE and 593 patients without AE), and 2 South Asian studies (43 patients with AE and 141 patients without AE). The characteristics of these studies are described in Table 2.

**Table 2:**
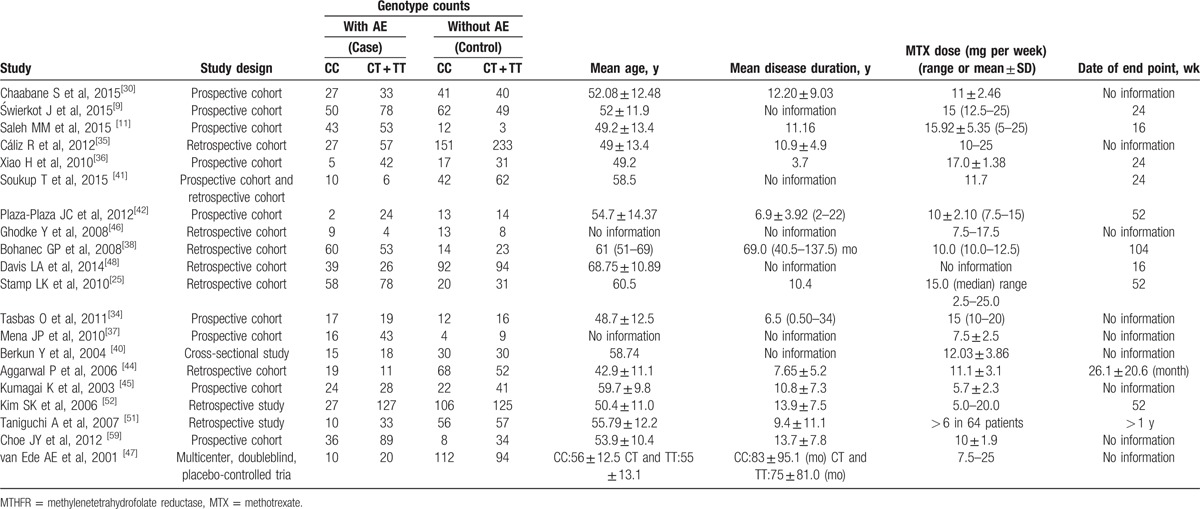
Summary of the analyzed studies and the distribution of methylenetetrahydrofolate reductase MTHFR 677C > T (rs1801133) genotypes.

**Figure 5:**
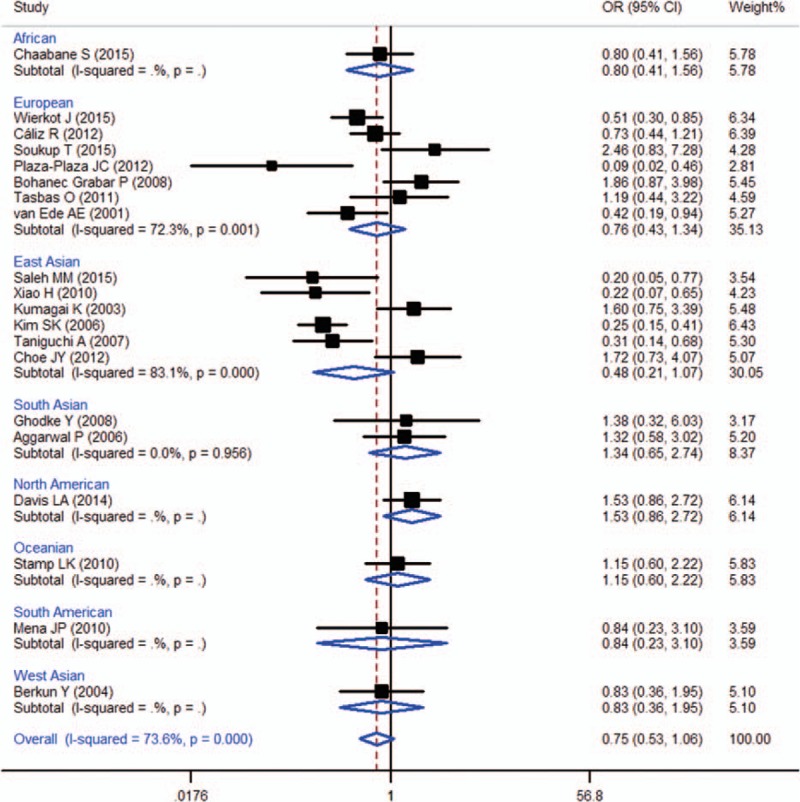
Meta-analysis of MTHFR 677C > T (rs1801133) single-nucleotide polymorphism and associated risk of toxicity of MTX (CC vs CT + TT genotypes). % weight = the percentage weight attributed to each study in the meta-analysis, CI = confidence interval, OR = odds ratio. Squares represent point estimates for effect size expressed as an OR with the size proportional to the inverse variance of the estimate. Lines represent 95% CIs. The diamonds represent the overall pooled estimate.

When all of the samples were included, the association between the frequency of 3 MTHFR 677C > T (rs1801133) alleles and MTX toxicity was not significant (OR = 0.75, 95% CI: 0.53–1.06, *Z* = 1.61, *P* = 0.107). Moreover, significant between-study heterogeneity was observed (*I*^2^ = 73.6%, *χ*^2^ = 71.86, *P* = 0.000) (Fig. 5).

Stratification by ethnicity did not identify a significant association between the MTHFR 677C > T (rs1801133) 3 allele frequency and MTX toxicity in the European (OR = 0.76, 95% CI: 0.43–1.34, *Z* = 0.94, *P* = 0.348), East Asian populations (OR = 0.48, 95% CI: 0.21–1.07, *Z* = 1.79, *P* = 0.074) or South Asian (OR = 1.34, 95% CI: 0.65–2.74, *Z* = 1.02, *P* = 0.309) (Fig. 5).

### 3.5 MTHFR 1298A > C (rs1801131)

Sixteen studies were included in the meta-analysis of MTHFR 1298A > C (rs1801131), which contained data from a combined total of 987 patients with AE and 1460 patients without AE and included 6 European studies (357 patients with AE and 783 patients without AE), 4 East Asian studies (266 patients with AE and 266 patients without AE). The characteristics of these studies are described in Table 3.

**Table 3:**
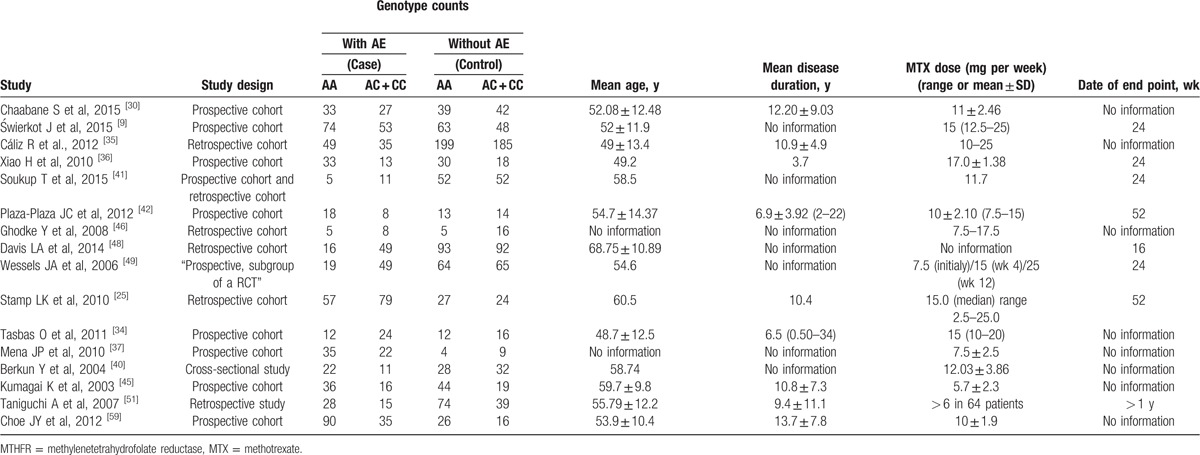
Summary of the analyzed studies and the distribution of methylenetetrahydrofolate reductase MTHFR 1298A > C (rs1801131) genotypes.

**Figure 6:**
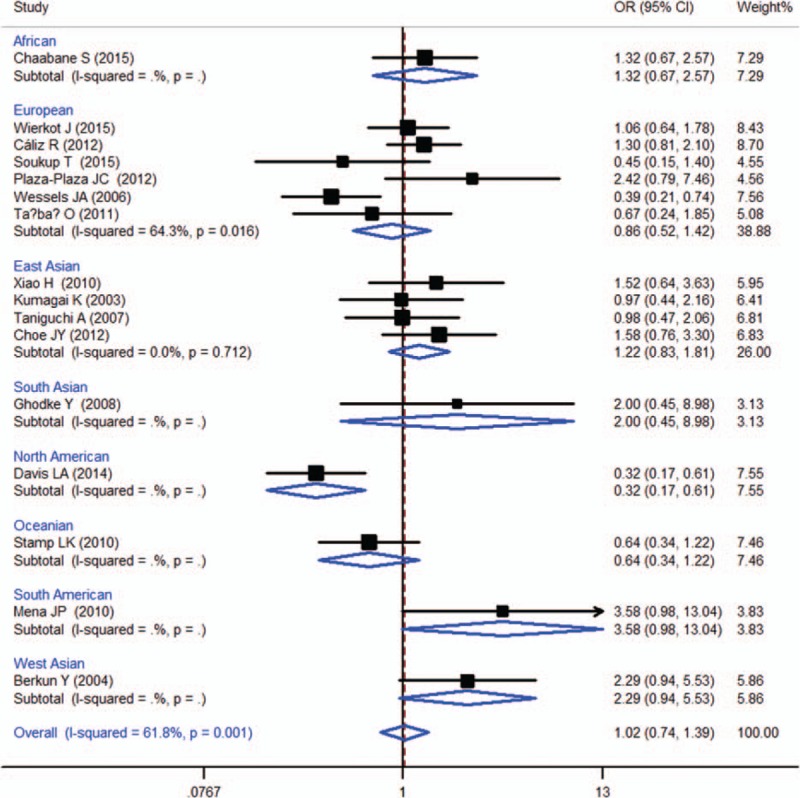
Meta-analysis of MTHFR 1298A > C (rs1801131) single-nucleotide polymorphism and the associated risk of of MTX (AA vs AC + CC genotypes). % weight = the percentage weight attributed to each study in the meta-analysis, CI = confidence interval, OR = odds ratio. Squares represent point estimates for effect size expressed as a OR with the size proportional to the inverse variance of the estimate. Lines represent 95% CIs. The diamonds represent the overall pooled estimate.

When all of the samples were included, the association between the MTHFR 1298A > C (rs1801131) 3 allele frequency and MTX toxicity was not significant (OR = 1.02, 95% CI: 0.74–1.39, *Z* = 0.10, *P* = 0.923). Moreover, significant between-study heterogeneity was observed (*I*^2^ = 61.8%, *χ*^2^ = 39.30, *P* = 0.001) (Fig. 6).

Stratification by ethnicity did not identify a significant association between the MTHFR A1298C (rs1801131) 3 allele frequency and MTX toxicity in the European (OR = 0.86, 95% CI: 0.52–1.42, *Z* = 0.59, *P* = 0.558), or the East Asian (OR = 1.22, 95% CI: 0.83–1.81, *Z* = 1.02, *P* = 0.309) (Fig. 6).

### 3.6 ATIC 347C > G (rs2372536)

Four studies were included in the meta-analysis of ATIC 347C > G (rs2372536), which contained data from a combined total of 311 patients with AE and 521 patients without AE. The characteristics of these studies are described in Table 4.

**Table 4:**
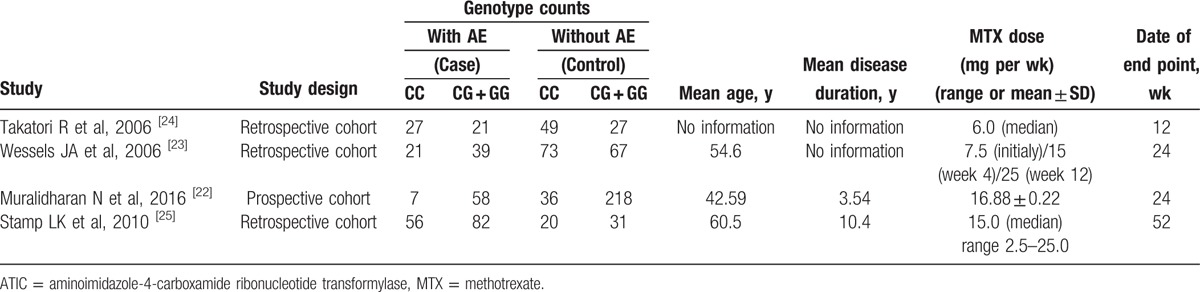
Summary of the analyzed studies and the distribution of methylenetetrahydrofolate reductase ATIC 347C > G (rs2372536) genotypes.

**Figure 7:**
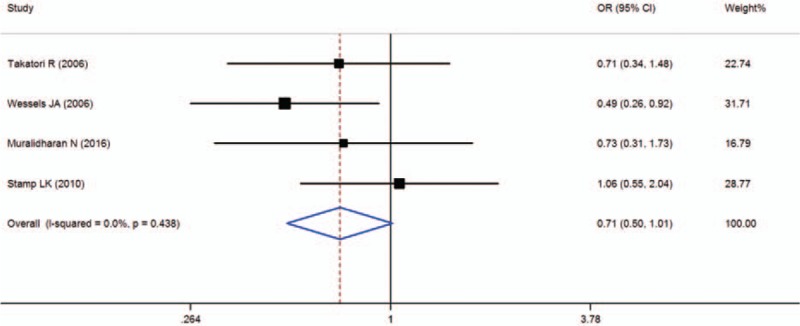
Meta-analysis of ATIC 347C > G (rs2372536) polymorphism and associated risk of toxicity of MTX (CC vs CG + GG genotypes). % weight = the percentage weight attributed to each study in the meta-analysis, CI = confidence interval, OR = odds ratio. Squares represent point estimates for effect size expressed as an OR with the size proportional to the inverse variance of the estimate. Lines represent 95% CIs. Diamond represents the overall pooled estimate.

When all of the samples were included, the association between the ATIC 347C > G (rs2372536) and MTX toxicity was not significant (OR = 0.71, 95% CI: 0.50–1.01, *Z* = 1.88, *P* = 0.060). Moreover, significant between-study heterogeneity was not observed (*I*^2^ = 0.0%, *χ*^2^ = 2.71, *P* = 0.438) (Fig. 7).

### 3.7 MTR 2756A > G (rs1805087)

Three studies were included in the meta-analysis of MTR 2756A > G (rs1805087), which contained data from a combined total of 228 patients with AE and 188 patients without AE. The characteristics of these studies are described in Table 5.

**Table 5:**
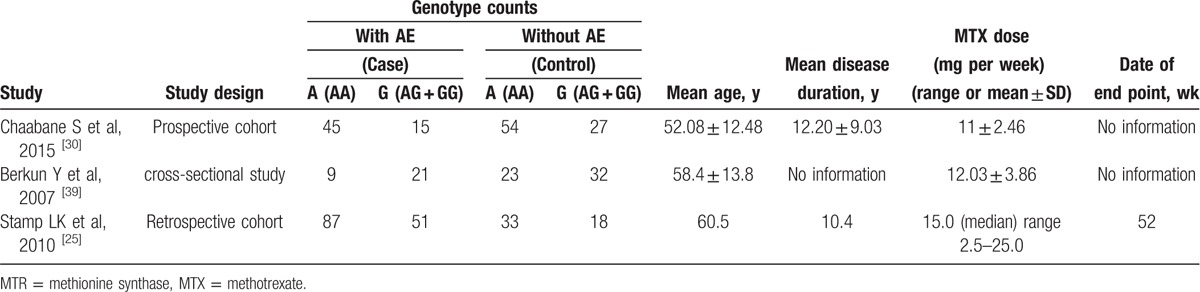
Summary of the analyzed studies and the distribution of methylenetetrahydrofolate reductase MTR 2756A > G (rs1805087) genotypes.

**Figure 8:**
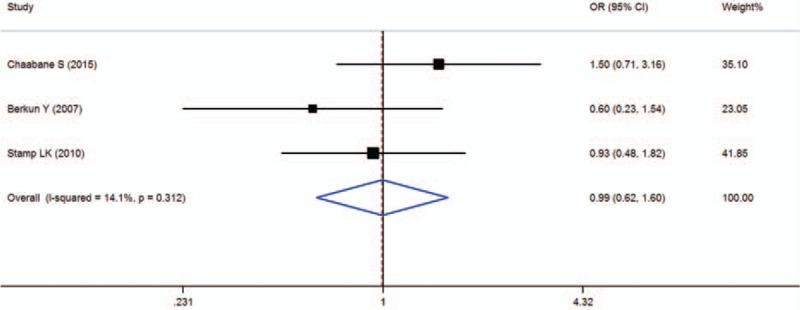
Meta-analysis of MTR 2756A > G (rs1805087) single-nucleotide polymorphism and associated risk of toxicity of MTX (AA vs AG + GG genotypes). % weight = the percentage weight attributed to each study in the meta-analysis, CI = confidence interval, OR = odds ratio. Squares represent point estimates for effect size expressed as a OR with the size proportional to the inverse variance of the estimate. Lines represent 95% CIs. Diamond represents the overall pooled estimate.

When all of the samples were included, the association between the MTR 2756A > G (rs1805087) allele frequency and MTX toxicity was significant (OR = 0.99, 95% CI: 0.62–1.60, *Z* = 0.03, *P* = 0.977). Moreover, significant between-study heterogeneity was not observed (*I*^2^ = 14.1%, *χ*^2^ = 2.33, *P* = 0.312) (Fig. 8).

### 3.8 MTRR 66A > G (rs1801394)

Two studies were included in the meta-analysis of MTRR 66A > G (rs1801394), which contained data from a combined total of 194 patients with AE and 132 patients without AE. The characteristics of these studies are described in Table 6.

**Table 6:**
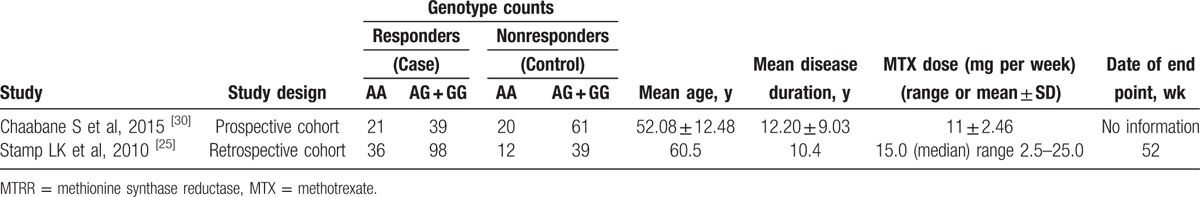
Summary of the analyzed studies and the distribution of methylenetetrahydrofolate reductase MTRR 66A > G (rs1801394) genotypes.

**Figure 9:**
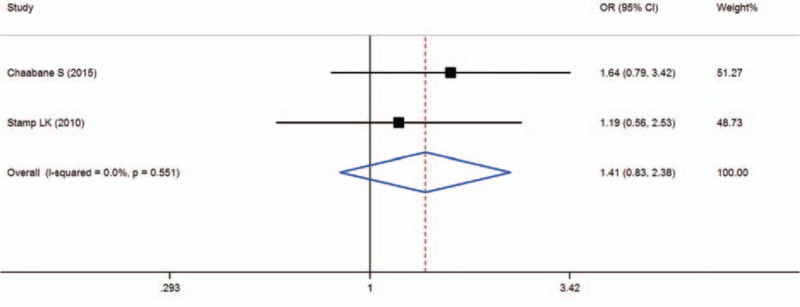
Meta-analysis of MTRR 66A > G (rs1801394) single-nucleotide polymorphism and associated risk of toxicity of MTX (AA vs AG + GG genotypes). % weight = the percentage weight attributed to each study in the meta-analysis, CI = confidence interval, OR = odds ratio. Squares represent point estimates for effect size expressed as a OR with the size proportional to the inverse variance of the estimate. Lines represent 95% CIs. Diamond represents the overall pooled estimate.

When all of the samples were included, the association between the MTRR 66A > G (rs1801394) allele frequency and MTX toxicity was not significant (OR = 1.41, 95% CI: 0.83–2.38, *Z* = 1.27, *P* = 0.203). Moreover, significant between-study heterogeneity was not observed (*I*^2^ = 0.0%, *χ*^2^ = 0.35, *P* = 0.551) (Fig. 9).

### 3.9 RFC −1 80G > A (rs1051266)

Ten studies were included in the meta-analysis of RFC -1 80G > A (rs1051266), which contained data from a combined total of 791 patients with AE and 1008 patients without AE and included 7 European studies (503 patients with AE and 865 patients without AE). The characteristics of these studies are described in Table 7.

**Table 7:**
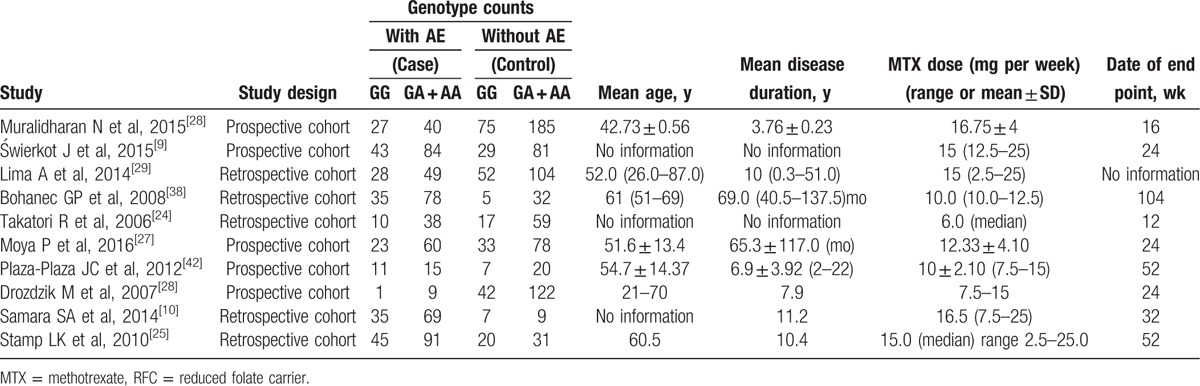
Summary of the analyzed studies and the distribution of methylenetetrahydrofolate reductase RFC −1 80G > A (rs1051266) genotypes.

**Figure 10:**
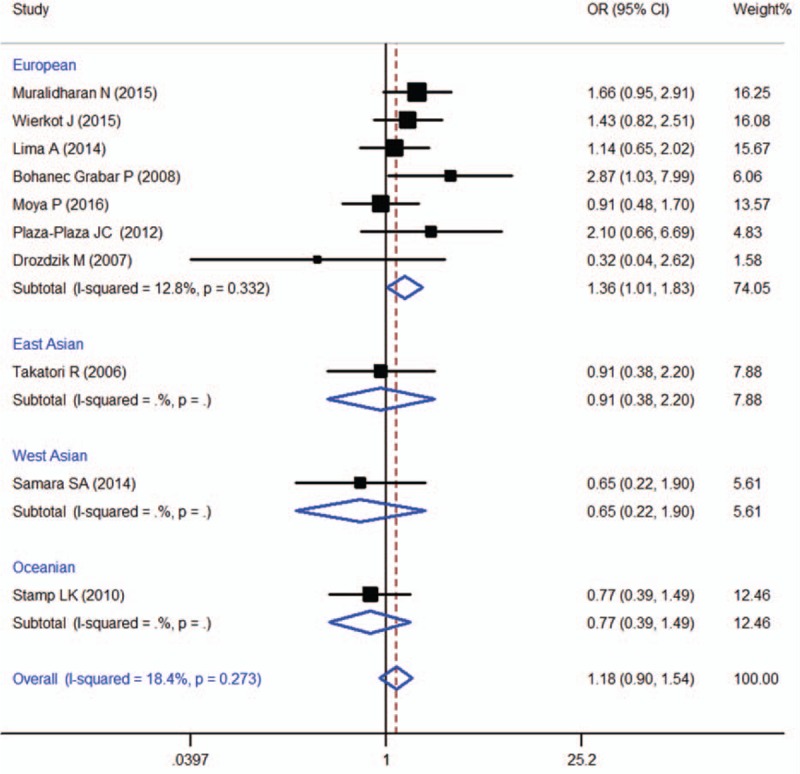
Meta-analysis of RFC-1 80G > A (rs1051266) single-nucleotide polymorphism and associated risk of toxicity of MTX (GG vs GA + AA genotypes). % weight = the percentage weight attributed to each study in the meta-analysis, CI = confidence interval, OR = odds ratio. Squares represent point estimates for effect size expressed as a OR with the size proportional to the inverse variance of the estimate. Lines represent 95% CIs. The diamonds represent the overall pooled estimate.

When all of the samples were included, no significant association was found between the RFC −1 80G > A (rs1051266) 3 allele frequency and MTX toxicity was identified (OR = 1.18, 95% CI: 0.90–1.54, *Z* = 1.21, *P* = 0.225). Moreover, significant between-study heterogeneity was not observed (*I*^2^ = 18.4%, *χ*^2^ = 11.04, *P* = 0.273) (Fig. 10).

Stratification by ethnicity identified a significant association between the RFC −1 80G > A (rs1051266) 3 allele frequency and MTX toxicity in Europeans (OR = 1.36, 95% CI 1.01–1.83, *Z* = 2.05, *P* = 0.041) (Fig. 10).

### 3.10 ABCB1 3435C > T (rs1045642)

Five studies were included in the meta-analysis of ABCB1 3435C > T (rs1045642), which contained data from a combined total of 391 patients with AE and 460 patients without AE. The characteristics of these studies are described in Table 8.

**Table 8:**
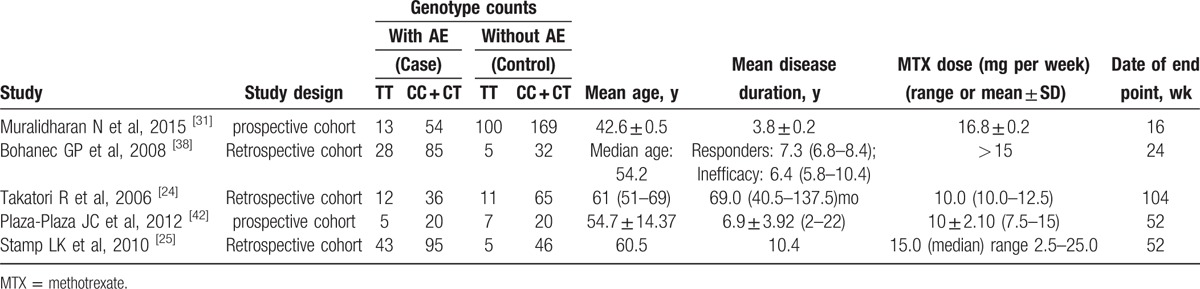
Summary of the analyzed studies and the distribution of methylenetetrahydrofolate reductase ABCB1 3435 C > T (rs1045642) genotypes.

**Figure 11:**
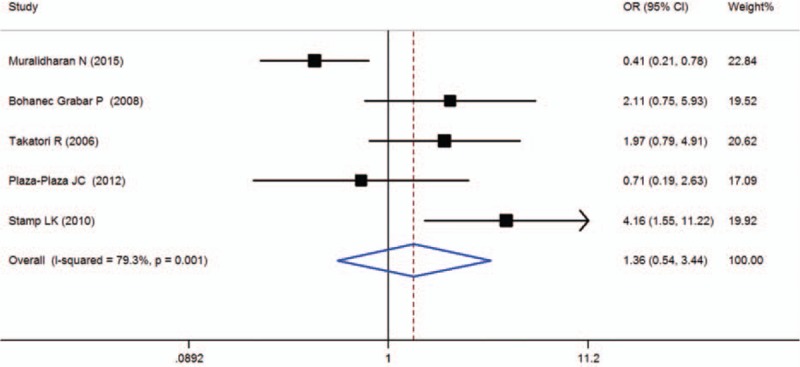
Meta-analysis of ABCB1 3435C > T (rs1045642) single-nucleotide polymorphism and associated risk of toxicity of MTX (TT vs CC + CT genotypes). % weight = the percentage weight attributed to each study in the meta-analysis, CI = confidence interval, OR = odds ratio. Squares represent point estimates for effect size expressed as a OR with the size proportional to the inverse variance of the estimate. Lines represent 95% CIs. Diamond represents the overall pooled estimate.

When all of the samples were included, no significant association between the ABCB1 3435C > T (rs1045642) 3 allele frequency and MTX toxicity was found (OR = 1.36, 95% CI: 0.54–3.44, *Z* = 0.65, *P* = 0.518). Moreover, significant between-study heterogeneity was not observed (*I*^2^ = 79.3%, *χ*^2^ = 19.32, *P* = 0.001) (Fig. 11).

## 4 Discussion

The pathogenesis of RA is not well understood, and there are considerable challenges in the design of effective medicines to cure RA. MTX is still the gold standard drug for RA and plays antiproliferative and anti-inflammatory roles in RA therapy.^[57,60]^ The toxicity of MTX comes to the most important factor in the failure of RA treatment. Although the factors influencing the toxicity in MTX remain unclear, genetic factors related to drug metabolism may play an important role in this variability. A single nucleotide polymorphism (SNP) is a common genetic variant that consists of a single DNA base pair change. The SNP association study has become a very popular method for identification of genetic factors for complex disease traits.^[61,62]^ Several studies have shown that SNPs could explain differences in genetic susceptibility to different diseases.^[63,64]^ In recent years, extensive pharmacogenomics investigations have been performed to optimize MTX therapy for RA patients through genotyping and/or gene-expression-based tests. These tests were primarily based on mRNA and included transporters, enzymes, and metabolites genes^[57]^; however, the majority of the findings were inconclusive and inconsistent, even for classical candidate gene polymorphisms. Thus, developing effective and practical biomarkers to aid in the prediction of MTX toxicity in routine clinical practice remains a challenge. The present study performed an SR on the association between polymorphisms and the toxicity of MTX in RA patients using papers published in the PubMed and Embase databases. Furthermore, this review focused on studies that reported the toxicity of MTX monotherapyand utilized pharmacogenetics, or the analysis of an individual's genetic variation, to predict the toxicity in MTX treated RA patients.

MTHFR is the most extensively studied MTX-related gene because it plays an important role in both responses and toxicity to MTX treatment in RA.^[14]^ MTHFR 677C > T (rs1801133) and 1298A > C (rs1801131) are 2 important polymorphisms that affect enzyme activity and MTX metabolism. MTHFR 677C > T is a nonsynonymous polymorphism that results in the substitution of alanine with valine at codon 222 of the MTHFR enzyme; MTHFR 1298A > C is another nonsynonymous polymorphism that leads to the substitution of glutamine with alanine in the C-terminal regulatory domain of the MTHFR enzyme, which results in decreased enzyme activity.^[58]^ In recent years, extensive investigations have been performed to identify the association between these 2 SNPs and MTX toxicity; however, the results were inconsistent. In the last decade, 4 meta-analyses were performed in relatively large samples, and the results suggested that the 2 SNPs were not associated with the toxicity of MTX in RA.^[4,13–15]^ The present study updated the meta-analysis, and a significant association was not observed between either the 677C > T (rs1801133) allele or the 1298A > C (rs1801131) allele and the MTX toxicity (OR = 0.75, 95% CI: 0.53–1.06, *Z* = 1.61, *P* = 0.107 and OR = 1.02, 95% CI: 0.74–1.39, *Z* = 0.10, *P* = 0.923, respectively). In addition, stratification by ethnicity did not identified a significant association between the MTHFR A1298C (rs1801131) 3 allele frequency and MTX toxicity in the European (OR = 0.86, 95% CI: 0.52–1.42, *Z* = 0.59, *P* = 0.558), and also did not identify a significant association between the MTHFR 677C > T (rs1801133) 3 allele frequency and MTX toxicity in the European (OR = 0.76, 95% CI: 0.43–1.34, *Z* = 0.94, *P* = 0.348) or East Asian populations (OR = 0.48, 95% CI: 0.21–1.07, *Z* = 1.79, *P* = 0.074).

ATIC is an important gene in the adenosine pathway, and it encodes an enzyme involved in the release of extracellular adenosine, which may have anti-inflammatory properties. Muralidharan et al^[22]^ reported that ATIC 347C > G gene polymorphism may be associated with the development of MTX induced gastrointestinal adverse events. Wessels et al^[23]^ also found that ATIC G allele carriers experienced a greater frequency of adverse events. However, a lack of association has been reported between the ATIC 347C > G gene polymorphism and the MTX toxicity.^[24,25]^ One meta-analysis found that the significant association between the ATIC 347 GG + GC genotype and MTX toxicity in Caucasians (OR = 1.741, 95% CI 1.080–2.806, *P* = 0.023), but not in Asian patients.^[16]^ In the present meta-analysis, when all of the samples were included, the association between the ATIC 347C > G (rs2372536) and MTX toxicity was not significant (OR = 0.71, 95% CI: 0.50–1.01, *Z* = 1.88, *P* = 0.060).

MTR and MTRR participate in folate metabolism and are also involved in the metabolism of adenosine. MTRR is an auxiliary factor of MTR and catalyzes the regeneration of the methylco-amine, maintains sufficient activation of MTR, and is indirectly involved in the process of in vivo methylation. The MTRR 66A > G gene polymorphism might affect the activity of the enzyme and the pharmacological effects of MTX. Dervieux et al^[26]^ observed that patients with A/A genotype at MTR 2756 and patients with G/G genotype at MTRR 66 had a significantly higher risk for gastrointestinal ADR than patients with MTR 2756G and MTRR 66A alleles. For the MTR A2756G (rs1805087) and MTRR 66A > G (rs1801394), 3 and 2 studies were included respectively in the present meta-analysis, but no significant association was observed between the 2 genotype and MTX toxicity.

Solute carriers, especially SLC19A1/RFC-1 and ABCs (ABCC1–4, ABCB1, and ABCG2) are 2 groups of MTX transporters that influence cellular MTX uptake and efflux. The RFC-1 80G > A (rs1051266), and ABCB1 3435C > T (rs1045642) polymorphisms were included in the present meta-analysis.

For RFC-1 80G > A (rs1051266), 10 studies with a total of 791 patients with AE and 1008 patients without AE were included in the present meta-analysis. When all of the samples were included, no significant association was found between the RFC-1 80G > A (rs1051266) 3 allele frequency and MTX toxicity was identified (OR = 1.18, 95% CI: 0.90–1.54, *Z* = 1.21, *P* = 0.225). Moreover, the stratification by ethnicity identified a significant association between the RFC -1 80G > A (rs1051266) 3 allele frequency and MTX toxicity in Europeans (OR = 1.36, 95% CI 1.01–1.83, *Z* = 2.05, *P* = 0.041). This result was inconsistent with a previous meta-analyses, which found that the RFC-1 80G > A polymorphism was not associated with toxicity to MTX therapy,^[1]^ and differences in the inclusion and exclusion criteria are the main reasons for these inconsistent conclusions. In the present study, we only focused on the association between gene polymorphisms and the toxicity to MTX monotherapy in RA patients and did not investigate gene–gene interactions.^[65]^ In addition, combined MTX and biologic disease-modifying anti-rheumatic drug (bDMARD) treatment^[66]^ were excluded from the meta-analysis of the RFC1 80G > A (rs1051266) polymorphism. Remarkably, the research from Lima et al^[67]^ was included in the present research but not in a previous meta-analysis because the same SNP (rs1051266) was identified by a different name (SLC19A1 G > A).

For the ABCB1 3435C > T (rs1045642) polymorphism, a previous meta-analysis that included 2 studies founded that MTX treatment toxicity was associated with the ABCB1 C3435T polymorphism in RA when an over-dominant model (TC vs TT + CC) was used (OR 0.483, 95% CI 0.259–0.900, *P* = 0.022), indicating that heterozygotes (TC) for the polymorphism had a lower risk for developing MTX toxicity than homozygotes (TT and CC).^[12]^ The present meta-analyses included 5 studies with 391 patients with AE and 460 patients without AE. When all of the samples were included, no significant association between the ABCB1 3435C > T (rs1045642) 3 allele frequency and MTX toxicity was found (OR = 1.36, 95% CI: 0.54–3.44, *Z* = 0.65, *P* = 0.518).

In addition to the above MTX transporter genes, an increased likelihood of toxicity has been reported to be associated withABCB1 SNPs, rs868755, rs10280623, and rs1858923.^[27]^ Stamp et al^[25]^ reported that there were weak associations between central nervous system adverse effects and AMPD1 34C > T (*P* = 0.04) and between gastrointestinal adverse effects and MTHFD1 1958G > A (*P* = 0.03) and ABCC2 IVS23 + 56T > C (*P* = 0.045), and there was a stronger association between any adverse effect and ABCG2 914C > A (*P* = 0.004). Lima et al demonstrated that SLC19A1, SLC46A1, and SLCO1B1 genotypes may help to identify patients with increased risk of MTX-related overall toxicity and that SLC19A1 and SLCO1B1 genotypes, and SLC19A1 haplotypes may help to identify patients with increased risk of MTX-related gastrointestinal toxicity.^[68]^

Certain limitations of our meta-analysis warrant consideration. First, the possibility of publication bias is always a concern. Although our analysis did not observe clear evidence of such a bias, it should be recognized that publication bias is difficult to exclude with certainty, especially when the number of incorporated studies is small. Second, publication bias could have distorted our meta-analysis because of the small number of included studies. We included 20, 16, 4, 3, 2, 10, and 5 studies in the meta-analysis of the MTHFR (677C > T (rs1801133) and 1298A > C (rs1801131)), ATIC 347C > G (rs2372536), MTR A2756G (rs1805087), MTRR 66A > G (rs1801394), RFC-1 80G > A (rs1051266), and ABCB1 C3435T (rs1045642) polymorphisms, respectively. Third, heterogeneity and confounding factors may have affected the meta-analysis. Variables such as sex, rheumatoid factor status, disease duration, and even patient's reports all have the potential to influence this analysis.

Taken together, this SR and meta-analysis demonstrated an association between MTX toxicity in RA patients and the RFC -1 80G > A (rs1051266) allele in European patients. Significant associations were not observed between the MTHFR (677C > T (rs1801133) and 1298A > C (rs1801131)), ATIC 347C > G (rs2372536), MTR 2756A > G (rs1805087), MTRR 66A > G (rs1801394), ABCB1 3435C > T (rs1045642), and RFC-1 80G > A (rs1051266, when all the patients were included) and the toxicity of MTX in RA patients. However, larger and more stringent study designs may provide more accurate results for the effect of these SNPs on the MTX treatment response.
